# Structure, Function and Inhibition of Helicases Involved in Virus Infection

**DOI:** 10.3390/biom16020273

**Published:** 2026-02-09

**Authors:** Gisoo Sarvari, David D. Boehr

**Affiliations:** Department of Chemistry, The Pennsylvania State University, University Park, PA 16802, USA

**Keywords:** viral helicases, viral gene expression, genome packaging, antiviral drug targets, helicase inhibitors

## Abstract

Viral helicases are conserved nucleic acid-dependent ATPases that drive genome replication, gene expression, and virion assembly, thereby playing a central role in viral replication and pathogenicity. Here, we discuss structural, biochemical, and virological data to compare helicase superfamilies, their conserved motifs, and translocation models that couple ATP hydrolysis to strand separation. We then analyze how viral helicases regulate replication fork progression, transcription and translation of viral RNAs, viral genome remodeling during replication, genome-packaging strategies, and evasion of innate immune signaling. Mechanistic examples from picornaviruses, flaviviruses, herpesviruses, and coronaviruses demonstrate how helicase architecture, substrate specificity, and cofactors control these activities. Finally, we discuss the opportunities and drawbacks of targeting viral helicases with antiviral drugs, recent screening and structure-guided discovery efforts, and emerging resistance mechanisms. Overall, this review provides a virus-centered synthesis of helicase structure, function, and inhibition that links conserved enzymatic activities to diverse infection outcomes and antiviral strategies across viral families.

## 1. Introduction

Helicases are important molecular motors that participate in the unwinding and remodeling of double-stranded (ds) nucleic acid structures such as DNA and RNA, using energy derived from ATP hydrolysis [1,2]. By coupling ATP hydrolysis to strand separation and translocation, helicases in general support multiple aspects of nucleic acid metabolism, including DNA replication, transcription, translation, recombination, repair, and RNA decay [3,4]. By unwinding nucleic acid strands, helicases enable access of polymerases and other key enzymes to their substrates, thereby supporting genome stability and regulated gene expression across these pathways.

### 1.1. Virus’ Dependence on Helicase Function

Viruses rely on helicases to replicate their genomes and coordinate key steps of the viral life cycle through genome unwinding, replication, and transcriptional regulation [3,4,5,6]. This dependence typically follows two primary strategies: viruses either encode their own helicases or recruit a host cell helicase [3,4]. For many RNA viruses, such as coronaviruses and flaviviruses, viral helicases are essential for the assembly of the replication-transcription complex [7,8]. For those viruses that lack their own helicases, like Human immunodeficiency virus type 1 (HIV-1), they hijack host helicases, including the DEAD-box helicase DDX3, to control the nuclear export and translation of their viral transcripts [3,4,9]. Severe viral diseases, including COVID-19, recurrent herpes simplex infections, and neurotropic flaviviral infections, are closely tied to efficient viral replication and immune evasion. In these cases, helicase activity affects viral load, persistence, and reactivation, with direct consequences for disease severity. This places viral helicases among the viral factors that directly influence disease outcome, rather than serving solely as background replication enzymes.

### 1.2. A Resurgence of Interest: Helicases as Therapeutic Targets and Immune Modulators

Viral helicases have long been considered as promising antiviral targets; previous progress includes the development of helicase inhibitors against Herpes simplex virus (HSV) [1,3]. The emergence of COVID-19 renewed interest in viral helicases as therapeutic targets [7,8] and recent advances in structural biology, computational modeling, and mechanistic studies of SARS-CoV-2 nonstructural protein 13 (nsp13), together with the high-throughput inhibitor screening, have enhanced the discovery of small-molecule inhibitors with potential antiviral activity and acceptable cytotoxicity [10,11]. Moreover, recent findings reveal that host helicases serve as essential components of innate immune sensing. This matters for drug discovery because viral helicases are direct targets, while targeting host helicases raises concerns with respect to selectivity and toxicity. These developments emphasize the urgent need to study viral helicases for their roles in infection and for their growing value as therapeutic targets [4].

In this context, viral helicases can be broadly distinguished based on substrate and functional context. DNA helicases primarily act on dsDNA during origin melting, replication fork progression, and genome packaging, whereas RNA helicases predominantly remodel structured single-stranded (ss) or duplex RNA to support genome replication, transcription, and translation; in both cases, strand separation typically proceeds via translocation along a single-stranded nucleic acid [12].

## 2. Classification and Structural Features

### 2.1. Helicase Superfamilies

**Figure 1:**
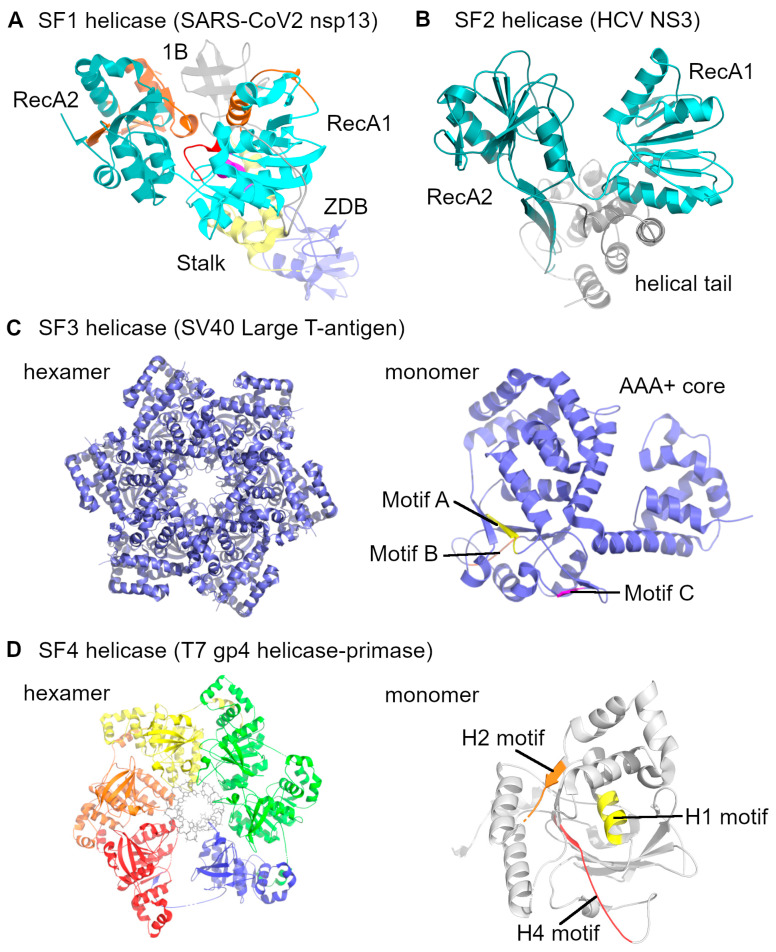
Structural comparison of domain architectures and oligomeric states across helicase superfamilies 1–4. Displayed are representative structures for each superfamily, highlighting the distinction between the monomeric helicases containing RecA-like cores (SF1, SF2) and the hexameric toroidal helicases (SF3, SF4). (**A**) Superfamily 1: Represented by the SARS-CoV-2 nsp13 helicase monomer [PDB: 6ZSL]. The structure is colored by domain, featuring the N-terminal Zinc-binding domain (ZBD, slate), Stalk (yellow), and 1B domain (orange) positioned above the two conserved RecA-like motor domains (RecA1, cyan; RecA2, teal). Conserved ATPase motifs are visualized as sticks: Walker A (red), Walker B (magenta), and other auxiliary motifs (orange). (**B**) Superfamily 2: Represented by the Hepatitis C Virus (HCV) NS3 helicase monomer [PDB: 1HEI]. The domain coloring distinguishes the conserved tandem RecA-like cores (RecA1, cyan; RecA2, teal) and the unique virus-specific C-terminal helical tail (grey). (**C**) Superfamily 3: Represented by the SV40 Large T-antigen [PDB: 1SVM]. Left: The functional hexameric ring assembly is displayed with subunits in slate blue. Right: A single monomeric subunit (slate blue) illustrates the AAA+ Core fold and the spatial arrangement of conserved motifs A, B, and C (shown in yellow, orange and magenta respectively). (**D**) Superfamily 4: Represented by the bacteriophage T7 gp4 helicase–primase [PDB: 6N7V]. Left: The hexameric helicase domain is shown encircling ssDNA (gray), with individual subunits colored distinctively. Right: A monomeric subunit highlights the RecA-like Core (light gray) and the characteristic H-family motifs (H1, H2, and H4/Arg Finger, shown in yellow, orange and red, respectively).

Helicases are essential to almost all nucleic acid processes, and their function is derived from two central structural folds, those being RecA-like domains and ATPases associated with diverse cellular activities (AAA+). Based on these structural folds, oligomeric form, conserved ATP-binding/hydrolysis motifs, and translocation direction, helicases are classified into six superfamilies (SF1 to SF6). SF1 and SF2 are monomers with two RecA-like domains, while SF3 to SF6 form hexamers or double hexamers consisting of RecA-like or AAA+ domains (see Figure 1 for representative structures). Helicases can be categorized by translocation polarity, with type A helicases moving along single-stranded DNA or RNA in the 3′→5′ direction, and type B helicases moving in the 5′→3′ direction. Regarding the substrate preference, α-helicases function on single-stranded DNA or RNA, while β-helicases are associated with dsDNA [13,14]. Virus-encoded helicases are found across multiple superfamilies, but are most commonly associated with SF1–SF3, whereas representatives of SF4–SF6 are less frequently observed in viral replication systems [1].

SF1. Superfamily 1 (SF1) helicases contain two RecA-like domains that form the ATPase active site together, while thirteen conserved motifs, including the Walker A (motif I), Walker B (motif II), and an arginine finger (motif IV) surround them. SF1 helicases are encoded by many positive-strand RNA viruses; for example, Tomato mosaic virus (ToMV) encodes an SF1 helicase domain within its replication protein [15]. In terms of the polarity of translocation, they are categorized into SF1A (3′→5′) and SF1B (5′→3′) enzymes. Viral SF1 helicases act on RNA during genome replication and associated steps of the viral life cycle [15].

SF2. Similar to SF1, superfamily 2 (SF2) helicases also have two RecA-like cores, but they are differentiated by the presence of an N-terminal Q-motif before Walker A, as well as at least nine more conserved sequence motifs (III–XII) [13]. Viral SF2 helicases are often processive DExH-type RNA helicases that bind to ssRNA and translocate in one direction to unwind duplex regions during genome replication. An example of this mechanism is hepatitis C virus (HCV) NS3, which separates strands mainly by “peeling” rather than by locally melting short duplexes, making it suitable for the extensive RNA remodeling required during viral replication [16].

SF3. Unlike SF1 and SF2, which were RecA-based helicases, superfamily 3 (SF3) helicases have a single AAA+-type core of approximately 100 amino acids [13]. SF3 helicases participate in the recognition of the origin site at the viral replication cycle, as well as in melting and unwinding [17]. Their function is coordinated by five motifs called A, B, B′ (unique insertion), C, and an arginine finger. These motifs control the ATP turnover at subunit interfaces. SF3 helicases typically assemble into a toroidal hexamer for function. They can also dimerize into a double hexamer under certain experimental conditions. This ring-shaped assembly can surround a single nucleic acid strand and move in the 3′→5′ direction and initiate the viral genome replication at specific origin sequences [14]. Papillomavirus E1, parvovirus Rep/NS1, and Simian virus 40 (SV40) large T antigen are representative members of SF3 helicases [17].

SF4. In the superfamily (SF4) helicases, the RecA-fold architecture is present, but the monomers are organized into a specialized six-subunit ring that surrounds the nucleic acid. Their function is coordinated by six signature motifs called H1, H1a, H2, H3, H4, and arginine motifs that organize the ATP hydrolysis and DNA binding. The hexamers translocate along the DNA duplex with the 5′→3′ polarity and unwind the DNA duplex. Bacteriophage T7 gp4, which carries an N-terminal primase domain, is a representative SF4 helicase [13,18].

SF5 and SF6. Superfamilies 5 and 6 (SF5–SF6) are mainly represented by cellular helicase systems rather than virus-encoded enzymes. SF5 is best exemplified by the bacterial transcription-termination factor Rho, a hexameric RecA-like ATPase that translocates on RNA in the 5′→3′ direction, whereas SF6 includes ring-shaped AAA+ motors involved in cellular DNA replication and genome maintenance (including MCM-family helicases and related AAA+ factors). Because SF5 and SF6 helicases are not commonly encoded by viruses, they are not a major focus of this review and are included only for classification completeness. More mechanistic information for SF5 and SF6 helicases can be gleaned from recent reviews [18,19].

### 2.2. Motifs in Helicases

Despite the variety of helicases, they share a set of conserved motifs that provide the ATP- and nucleic acid–binding sites required for activity. While dispersed along the primary sequence, these motifs assemble within the folded protein and form the catalytic core [20,21]. In SF1 and SF2 helicases, the conserved set of motifs comprises I, Ia, Ib, II, III, IV, V, and VI [20]. Walker A (motif I; GxxxxGKT/S) mediates ATP binding through a conserved lysine, and Walker B (motif II; DEAD/DEAH) coordinates Mg^2+^ via a conserved aspartate, both of which are essential for ATP hydrolysis in viral helicases [20,21]. Motif III acts as a coupling element between ATP hydrolysis and strand separation, and SAT box mutations eliminate strand unwinding while keeping ATP hydrolysis intact [20]. Motifs IV and V interact with the phosphate backbone and nucleic acid binding; motif VI functions as a bridge between the ATP- and nucleic acid–binding sites, inducing the conformational changes that couple ATP hydrolysis with nucleic acid binding and unwinding [21]. In many viral SF2 helicases, a conserved Q-motif, is positioned upstream of Walker A towards the N-terminus. Although originally characterized using DEAD-box prototypes, this motif is also conserved in viral SF2 helicases, where it contributes to efficient ATP utilization during RNA genome replication [21]. In SF3 helicases, three conserved motifs, A, B, and C, are present, along with an additional B′ motif located between motifs B and C [2]. Their Walker B variant (XXXXEE) is different from the typical DEAD/DEAH sequence [21]. Motif C has a conserved asparagine that hydrogen-bonds with the γ-phosphate of ATP and enables the helicase to distinguish ATP from ADP [20]. B′ motif is a glycine and lysine-rich sequence that was identified more recently and is important for ATP hydrolysis and strand unwinding [22]. SF4 helicases have conserved motifs (H1–H4) clustered in the C-terminal region that form a catalytic core [13,18]. Altogether, helicases across different superfamilies share a core set of conserved motifs; however, they can also have unique motif variants to facilitate virus genome replication.

### 2.3. Mechanistic Models of Nucleic Acid Unwinding

#### 2.3.1. Inchworm Model

**Figure 2:**
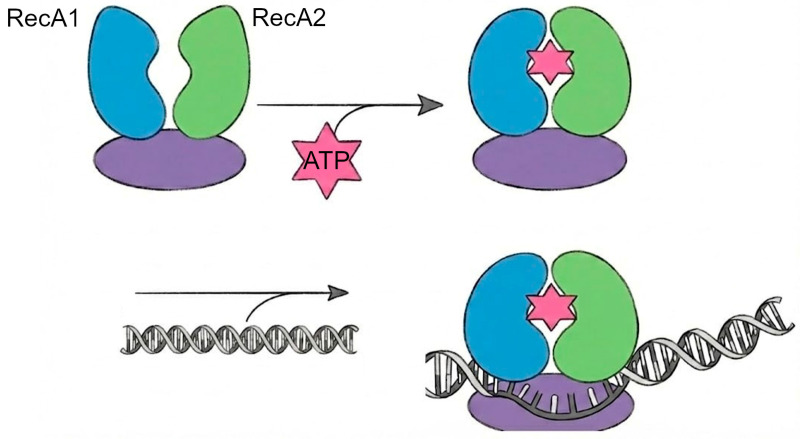
ATP-dependent alternating movements of two RecA-like domains drive stepwise translocation along nucleic acid. Structural elements are depicted schematically and are not drawn to scale.

Monomeric viral helicases, such as Coronavirus nsp13 and flavivirus NS3, are proposed to function via an inchworm mechanism (Figure 2) [23]. In this model, directional translocation results from coordinated conformational changes between the two RecA-like domains that form the helicase core. Structural and single-molecule studies on SF1 helicases indicate that nucleotide binding and hydrolysis at the domain interface cause alternating shifts between the two RecA-like domains while the helicase remains bound to the nucleic acid [24]. Conformational changes shift interactions with the tracking strand, leading to stepwise translocation along single-stranded nucleic acid. Duplex separation occurs at the ss–ds junction through conserved structural elements, such as β-hairpins, that destabilize the duplex during forward movement [25]. Despite the limited high-resolution mechanistic work on viral helicases, current structural and biochemical evidence supports the idea that viral helicases use this conserved RecA-domain–coupled inchworm mechanism during genome replication. In coronaviruses, nsp13 operates as part of the replication–transcription complex, coordinating helicase translocation with RNA synthesis [23]. Similarly, flavivirus NS3 appears to rely on the same kind of RecA-domain motions to unwind RNA during replication [26].

#### 2.3.2. Sequential Staircase or Hand-over-Hand Model

**Figure 3:**
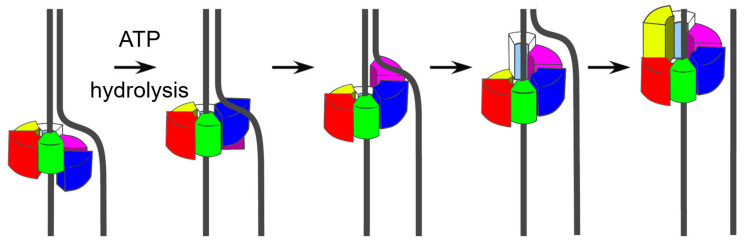
Staircase or hand-over-hand translocation model of hexameric viral helicases: Sequential ATP hydrolysis (indicated by arrows) around the hexamer drives stepwise movement of subunits that form a spiral staircase around the DNA strand. Each subunit releases and rebinds further along the backbone, advancing the nucleic acid through the central pore without ring rotation. This mechanism underlies the high-processivity unwinding activity of SF3 and SF4 helicases such as SV40 large T antigen, papillomavirus E1, and bacteriophage T7 gp4.

In hexameric viral helicases such as SV40 large T antigen, bacteriophage T7 gp4, and papillomavirus E1, nucleic acid unwinding is proposed to occur through a sequential translocation mechanism that requires the coordinated function of six subunits (Figure 3). Structural studies indicate that each subunit contributes a DNA-binding loop to the central channel, and these loops collectively form a spiral staircase around the tracking strand [27]. As ATP is hydrolyzed sequentially around the ring, subunits disengage in turns and step forward to the opposite end of the staircase. This mechanism drives the DNA strand through the pore with high processivity but requires precise coordination, since even the loss of a single ATPase site in T7 gp4 is enough to stop translocation [28]. This mode of translocation is commonly referred to as the staircase mechanism based on its structural organization; the same process has also been described as a hand-over-hand or escort model, reflecting its sequential ATPase cycling and loop-mediated strand engagement. Step size is different between helicases: In papillomavirus E1, which is an SF3 viral helicase, each subunit interacts with one nucleotide, adding up to a net advance of ~6 bases per ATPase cycle; while in gp4, each subunit engages two nucleotides, adding up to a net advance of ~12 bases per ATPase cycle [25]. Biochemical assays showed both 1-nt and 2-nt steps, but, according to structural studies, the actual step size is defined by the number of nucleotides engaged by each subunit.

Hexameric helicases have various overall shapes. In SF4 helicases, such as T7 gp4, the hexamer forms a lockwasher configuration, where the two ends of the ring are staggered relative to one another; this gap is connected by flexible N-terminal linkers. By contrast, AAA+ helicases such as E1 (SF3) and SV40 large T antigen (SF3/SF6) assemble into symmetric closed rings [25,27]. Cryogenic electron microscopy (Cryo-EM) studies of T7 gp4 support the hand-over-hand mechanism [29]. The stepwise progression of subunits accounts for the high processivity of hexameric viral helicases and enables them to unwind long dsDNA genomes, such as those of SV40 and papillomaviruses.

## 3. Functional Roles in Infection

### 3.1. Genome Replication, Fork Progression and Strand Separation

This section builds on the structural and mechanistic descriptions in Section 2 to address how helicase activity is coupled to viral genome replication, replication fork progression, and strand separation in infected cells. High-resolution cryo-EM studies show how hexameric viral helicases (SF3–SF6) use energy from ATP hydrolysis to perform directional strand separation through a conserved “staircase” mechanism (see Figure 3) [30]. In the SV40 large T antigen, six DNA-binding loops form a pseudo-hexameric spiral that alternately grips phosphates of the tracking strand after another. As the helicase moves forward, part of the protein called the “wedge domain” pushes the complementary strand through a side opening. The ATP cycle proceeds in a sequential manner: ATP binds and causes a loop to hold the DNA, and then ATP is hydrolyzed, which weakens the grip, and the phosphate is released to reset the cycle for the next step [31]. A similar strategy operates in bacteriophage T7 gp4, which also forms a hexameric ring around the lagging-strand template and moves 5′→3′ while its primase domain synthesizes RNA primers for Okazaki fragment initiation [9].

By contrast, SF1 helicases, such as the coronaviral nsp13, translocate 5′→3′ on RNA and combine strand unwinding with additional functions that help set up downstream processes. An N-terminal zinc-binding domain (ZBD) first binds to dsRNA at the 5′ end to initiate the process and catalyzes a 5′-triphosphatase reaction that provides substrates for viral capping. High-resolution cryo-EM structures of SARS-CoV-2 nsp13 show five primary elements, ZBD, stalk, 1B, 1A, and 2A that move back and forth (“rock”) sequentially during each ATPase cycle. In an inchworm-like step, ATP binding causes domains 1A and 2A to shift from open to closed, pulling apart base pairs and threading the tracking strand into a central channel. The other strand exists via a side opening near the ZBD [11,32]. By coordinating a ring-shaped structure with ATPase cycles, these helicases convert chemical energy into rapid (hundreds of bases per second) mechanical unwinding. This function lays the molecular foundation for both continuous leading-strand synthesis and discontinuous lagging-strand replication.

### 3.2. Transcriptional Regulation by Viral Helicases

Viral helicases use ATP-dependent remodeling of nucleic acids to regulate gene expression during transcription. Initially, during this stage, viral helicases unwind and rearrange DNA and RNA substrates to facilitate the binding and function of RNA polymerase and productive elongation. Specifically, helicases unwind promoter regions or nascent viral RNA to expose single-stranded templates, remove RNA-DNA hybrids, and restart blocked transcription complexes, thereby supporting efficient synthesis of viral transcripts [18,33]. For instance, the SV40 large T antigen hexamer binds the initial DNA and employs the same ATP-driven staircase hand-over-hand mechanism to melt duplexes, an important step in initiating viral transcript synthesis [1,5]. During viral transcription, helicases function within virus-specific transcription or replication–transcription complexes, where they coordinate nucleic-acid remodeling with viral polymerases and associated factors. Disruption of these helicase-mediated interactions compromises viral RNA synthesis and transcriptional progression, leading to inefficient gene expression and impaired viral replication [3,4].

### 3.3. Genome Packaging and Assembly

**Figure 4:**
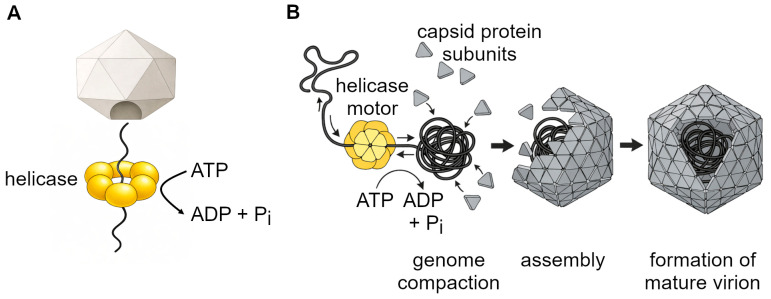
Viral genome packaging and assembly mechanisms: (**A**) packaging into pre-assembled capsids mediated by a hexameric helicase that utilizes ATP hydrolysis to translocate the viral genome through a portal into the capsid, (**B**) co-assembly of genome and capsid, in which the helicase progressively compacts the nucleic acid while capsid subunits assemble around the condensed genome to form the mature virion.

After viral genome replication, the newly synthesized nucleic acids must be packaged into new capsids. This process can also be powered by viral helicases, which can convert ATP energy into mechanical force to either pull the genome into an existing capsid or compact it within an assembling capsid (Figure 4). Here, the term genome packaging helicases is used as a functional description for viral helicases involved in genome encapsidation, rather than a distinct helicase subfamily. Despite mechanistic differences across viral families, genome packaging helicases share several common features. First, the involved helicases usually assemble into ring-like oligomers, typically hexamers, that surround a single strand of nucleic acid. Second, the activity of helicases is based on ATP binding and hydrolysis that induce conformational changes during each cycle and lead to the stepwise translocation or compaction of the viral genome. Third, packaging helicases can detect specific sequences or structural motifs within viral DNA/RNA to ensure the selective packaging of only the viral genome. Finally, genome packaging helicases work together with the capsid proteins. Some viruses bind to a specific opening in the preassembled capsid and insert the genome into the capsid. In other viruses, the helicases compact the nucleic acid so that the capsid proteins can assemble around it. In general, there are two main genome-packaging strategies: packaging into preformed capsids and coassembly of the genome and capsid [34].

#### 3.3.1. Packaging into Pre-Formed Capsids (dsDNA Viruses)

In many dsDNA viruses, such as bacteriophage λ, the prohead, which is an empty protein shell, is assembled first. Subsequently, a multi-protein complex called terminase attaches to the viral DNA, cuts it at specific recognition “cos” sequences, and then docks at a portal in the capsid [35]. Using ATP hydrolysis as its power source, the terminase pulls hundreds of base pairs of DNA into the capsid against the internal pressure. Once the headful signal occurs and the whole genome is inside the capsid, terminase cuts the DNA again, releases it, and the capsid seals to form an infectious virion [36].

#### 3.3.2. Packaging into Pre-Formed Capsids (ssDNA Viruses)

Adeno-associated virus (AAV) is a good example of a virus that packages its ssDNA into a pre-assembled capsid through a two-step process. A family of non-structural proteins called Rep is involved with this process. First, the Rep78/68 proteins that are the larger isoforms, cut the viral DNA at its terminal repeat sequences, which creates a free 3′ end that the Rep52/40 helicase can attach to. Next, six Rep52/40 helicase subunits encircle the free genome end. In each cycle of ATP binding and hydrolysis, one Rep52/40 subunit remains attached to the DNA and holds it in place, while the remaining continue moving, allowing the helicase to move stepwise through the strand and produce a sequential stepping mechanism to pull the genome into the capsid [37].

#### 3.3.3. Packaging Segmented Double-Stranded RNA (φ6 Bacteriophage)

Bacteriophage φ6 packages its three dsRNA genomes into a single capsid. The P4 ATPase assembles as a hexamer, specifically binds to the 5′ ends of each +RNA segment, and moves them into the procapsid via ATP hydrolysis. During each translocation, conformational changes inactivate the current binding site, exposing the next one. This stepwise, ordered mechanism ensures that the entire genome, including the S, M, and L components, fits properly prior to virion maturation [38].

#### 3.3.4. Co-Assembly of Genome and Capsid (ssRNA Viruses)

Certain ssRNA viruses assemble the capsid around the genome simultaneously rather than placing them in a preformed shell. Helicases such as alphavirus nsP2 or flavivirus NS3 are good examples; they interact with RNA to compact it, remodel stem-loops, and facilitate the engagement of capsid proteins. While the RNA is being clustered, the capsid proteins assemble around it, forming a highly packed infectious virion [39].

### 3.4. Viral Helicases and Immune Evasion

In addition to genome replication, viral helicases play a significant role in interfering with host innate immune responses to viruses. Helicases achieve immune evasion via three primary mechanisms: RNA remodeling to avoid detection, direct antagonism of sensors and adaptor proteins, and subcellular shielding, which hides immunogenic molecules within subcellular compartments.

#### 3.4.1. Removal or Remodeling of Immunostimulatory RNA

During viral replication, helicases usually process newly formed RNA duplexes or triphosphorylated 5′ ends to remove pathogen-associated molecular patterns (PAMPs) that would typically trigger host cytosolic RNA- sensing pathways. For example, in the West Nile (WNV) virus, the NS3 helicase functions within membrane invaginations to unwind replication-derived dsRNA. This reduces the availability of blunt-ended duplexes in the cytoplasm and, consequently, limits innate immune activation [40]. There are two helicase-associated decapping enzymes called D9 and D10 in poxviruses that selectively eliminate the 5′ cap from viral mRNAs, hindering the buildup of uncapped RNAs that result in the activation of host RNA sensors [41]. Similarly, the coronavirus nsp13 helicase partners with the viral capping complex to ensure complete methylation of the RNA cap, masking the 5′-triphosphate signature and evading host RNA sensor detection [42].

#### 3.4.2. Direct Antagonism of Sensors and Adaptor Proteins

Some viral helicases or helicase-related proteases directly bind and inhibit host RNA-sensing receptor components or their downstream adaptors, functioning as competitive inhibitors. For example, the HCV NS3 helicase–protease complex cleaves Mitochondrial antiviral signaling protein (MAVS) at the mitochondrial outer membrane, disrupts downstream signaling to antiviral transcription factors, and blocks interferon-β induction [43]. In picornaviruses, the 2A and 3C proteases, often in complex with viral helicase domains, disrupt innate immune signaling by cleaving MAVS and the TLR3 (Toll-like receptor 3) adaptor TIR-domain-containing adapter-inducing interferon-β (TRIF), thereby concurrently shutting down the RNA sensing and TLR3 pathways [43]. Influenza A virus takes a non-proteolytic approach: the PA subunit, which harbors helicase-like motifs, binds and sequesters the RIG-I coactivator PACT (Protein kinase R activating protein), thereby blocking ATPase-dependent activation of cytosolic RNA sensing without degrading any host proteins [44].

#### 3.4.3. Subcellular Relocalization and Replication Compartment Shielding

Numerous positive-strand RNA viruses remodel host cell intracellular membranes into replication organelles to help shield the replication complex from being detected by the cytosolic immune sensors [45]. Alphaviruses remodel plasma membranes into double-membrane spherules using their nsP2 helicase [46]; these specialized compartments concentrate replication intermediates while limiting exposure to host RNA sensors. The Vaccinia virus uses its A18 helicases in cooperation with the K7 protein to produce atypical stress granules that trap DDX3X, hindering its assembly with downstream antiviral signaling kinases and subsequently reducing Interferon regulatory factor 3 (IRF3) activation [47].

Altogether, these mechanisms demonstrate how viral helicases and their related proteins disrupt host RNA signaling to defeat innate immunity. Together, they limit detection and downstream signaling while supporting efficient replication.

## 4. Examples by Virus Family

Hundreds of viral helicases are known; however, only a few have been studied sufficiently to demonstrate the main mechanical principles described above. Here, each example serves as a case study to illustrate (1) preferences for RNA or DNA substrates, (2) inchworm versus staircase translocation, and (3) functional coupling to partner domains or cofactors (see Table 1 for summary). Overall, these examples indicate that helicase-mediated processes influence viral replication efficiency and pathogenic outcomes, supporting their relevance as targets for antiviral intervention.

### 4.1. Picornaviruses

The picornaviral 2C protein is predicted to be an SF3/AAA+ P-loop NTPase and has long been discussed as a helicase candidate. Despite the NTPase activity observed in the purified poliovirus 2C, no definitive duplex-unwinding activity has been observed in vitro. In other words, ATP hydrolysis by 2C may facilitate replication through mechanisms beyond classical duplex unwinding, such as remodeling RNA–protein assemblies or replication organelles. However, the exact role is unresolved. If 2C operates as a ring ATPase, it would likely translocate through the staircase mechanism as described for other SF3 hexameric enzymes. This 2C example shows that ATP hydrolysis and complex remodeling do not necessarily prove strand-unwinding activity of 2C [48,49,50]. Despite uncertainty regarding its precise mechanistic role, poliovirus 2C is required for viral replication and contributes to the neuropathology of poliomyelitis. Poliovirus relevance persists in the post-eradication era, particularly regarding persistent infection and viral shedding in immunocompromised individuals [51].

### 4.2. Flaviviruses

Flaviviral NS3 is a well-characterized SF2 RNA helicase, located N-terminal to a chymotrypsin-like serine protease (NS3pro). NS4A activates the protease and, along with replication-membrane cofactors such as NS4B, provides functional support. The helicase core is composed of two RecA-like domains that form an interdomain cleft, which coordinates NTP and RNA binding, a defining feature of SF1 and SF2 enzymes [1]. Structural and biochemical studies of RecA-fold helicases show that they translocate through an inchworm mechanism, in which ATP binding and hydrolysis drive alternating grip-and-release movements of the two domains, advancing the enzyme by one nucleotide per cycle. NS3 is the viral example of this process, linking its helicase activity to the protease domain to coordinate RNA remodeling with polyprotein processing at replication membranes [52]. NS3 contributes to flaviviral pathogenesis through its combined protease and helicase functions, which are required for genome replication and virion production in clinically relevant flaviviruses, including dengue virus, Zika virus, West Nile virus, and yellow fever virus [53]. Interference with NS3-mediated RNA remodeling or polyprotein processing impairs viral replication and is associated with reduced disease severity, supporting its relevance as an antiviral target [54].

### 4.3. Herpes Simplex Virus (HSV)

HSV assembles a helicase-primase complex consisting of UL5, UL52, and UL8 that is essential for viral DNA replication. UL5 is the helicase subunit; it has the conserved SF1 NTPase/helicase motifs and provides 5′→3′ unwinding activity. UL52 functions as the primase, providing the primase active site and a zinc finger that enables direct DNA contacts. UL8 is a nonenzymatic cofactor that enhances both enzymatic activities, facilitates nuclear transport of the complex, and helps coordinate replication with other viral proteins. The helicase-primase requires ATP and the viral ssDNA-binding protein ICP8 to unwind duplex DNA. Mutational studies show that the SF1 motifs in UL5 are critical for helicase activity and that UL52 directly contacts DNA while UL5 functions near the replication fork [55]. The helicase–primase complex is required for lytic replication and viral spread in epithelial and neuronal tissues, linking its activity to recurrent mucocutaneous disease and severe outcomes such as neonatal herpes and herpes encephalitis [56]. Efficient helicase–primase function is also necessary for sustained viral replication and repeated reactivation from neuronal reservoirs, contributing to HSV pathogenesis [57]. As this complex is virus specific and distinct from host replication machinery, it provides a defined viral helicase system with direct pathological relevance and a clear rationale for therapeutic targeting.

### 4.4. Coronaviruses

There is no DNA present in the replication cycle of the coronaviruses, such as SARS-CoV; however, nsp13 helicase can unwind both RNA and DNA duplexes in the 5′→3′ direction using ATP hydrolysis or any of the eight classical NTPs [58]. Additionally, nsp13 shows RNA 5′-triphosphatase (RTPase) activity, cleaving the terminal phosphate from the 5′ end of RNA as part of RNA capping. It was observed that ATP competitively inhibits RTPase activity, suggesting that the active sites of helicase and RTPase overlap [59]. In vitro assays have confirmed these properties, and DNA unwinding is probably irrelevant in infection. Recent studies indicate that nsp13 has a conserved SF1/SF2 helicase-type structure, consisting of a RecA-like core, 1B insert, stalk, and ZBD, and it moves through an ATP-driven inchworm mechanism. The druggability and mechanistic insights of nsp13 in the replication–transcription complex (RTC) have been confirmed by fragment screening around the ATP and RNA channels [7,60]. Nsp13 is required for coronavirus replication and is conserved across pathogenic coronaviruses, linking its helicase and RNA-capping functions to viral fitness and disease severity in SARS, MERS, and COVID-19. By supporting efficient viral RNA synthesis, nsp13 contributes to the high viral loads associated with severe respiratory disease and systemic manifestations in pathogenic coronavirus infection [61]. Altogether, Its essential role, conservation across pathogenic coronaviruses, and multifunctional activities position nsp13 as a promising target for broad-spectrum antiviral development [62].

**Table 1:** Comparative Table of Helicases.

Virus Family	Helicase Protein	Superfamily (SF)	Substrate (RNA/DNA)	Directionality	Unwinding Mechanism	Other Notes
Positive-sense single-stranded RNA viruses						
Coronaviridae e.g., SARS-CoV, SARS-CoV-2, MERS-CoV	nsp13	SF1B	RNA & DNA	5′→3′	Inchworm	Also 5′-triphosphatase; central to replication–transcription complex [7]; PDB ID: 6JYT
Flaviviridae e.g., HCV, Dengue, WNV, JEV	NS3	SF2	RNA	3′→5′	Inchworm (monomer/dimer)	Dual protease–helicase; requires NS4A/NS4B [12,63]; PDB ID: 1A1V
Picornaviridae e.g., PV	2C	SF3 (AAA+ hexamer)	RNA	3′→5′	Staircase (hexameric)	Membrane remodeling for replication vesicles; helicase activity debated [12,48,49]; PDB ID: 5Z3Q
Single-stranded DNA virus						
Parvoviridae e.g., AAV2	Rep52/40	SF3	DNA	3′→5′	Staircase (hexameric)	Packages ssDNA genomes into capsids; Rep78/68 required for initiation [12,64]; PDB ID: 1S9H
Double-stranded DNA viruses						
Bacteriophage T4	gp41	SF4 (DnaB-like)	DNA	5′→3′	Staircase (hexameric)	Works with gp59 loader and gp61 primase; classic replication fork model [65]; PDB ID: 4LJY
Bacteriophage T7	Gene 4 helicase–primase	SF4 (DnaB-like)	DNA	5′→3′	Staircase (hexameric)	Bifunctional primase–helicase; coordinates fork progression [28,66]; PDB ID: 1E0J
Cystoviridae e.g., Phage φ6	P4	SF4-like	RNA	5′→3′	Staircase (hexameric)	Packaging motor for dsRNA genome into procapsid [38,67]; PDB ID: 1W44
Herpesviridae e.g., HSV-1/2	UL5 (with UL8/UL52)	SF1/SF3 hybrid	DNA	5′→3′	Inchworm-like	Helicase–primase complex; coordinates unwinding with priming [55]
Papillomaviridae e.g., HPV18	E1	SF3	DNA	3′→5′	Staircase (hexameric)	Binds replication origin with E2; initiates DNA melting [68]; PDB ID: 2GXA
Polyomaviridae e.g., SV40	Large T antigen	SF3	DNA	3′→5′	Staircase (hexameric)	Origin-binding domain + helicase; model dsDNA replicative helicase [12,69]; PDB ID: 1SVM
Poxviridae e.g., Vaccinia	NPH-II	SF2	RNA	3′→5′	Inchworm	Required for viral transcription; RNA remodeling and decapping [16,70]

## 5. Antiviral Targeting of Helicases

### 5.1. Why Helicases Are Druggable

Helicases are among the most conserved and critical viral enzymes, playing important roles at various steps of the viral lifecycle, including genome replication, transcription, translation, and packaging. The lack of viral helicases leads to blocked polymerases at secondary structures, collapsed replication forks, and inefficient processing of viral RNA, all of which make helicases an important drug target [3,31]. There are specific catalytic regions in the structure of helicases that can readily bind to small molecules. The conserved domains for ATP binding, such as the Walker A/B motifs, the arginine finger, and the nucleic acid-tracking channels, are readily accessible pockets for other molecules to bind [32,71]. As discussed in Section 4, helicase-driven processes are directly linked to disease-relevant replication outcomes across multiple virus families, and these same systems have yielded direct-acting helicase inhibitors spanning biochemical probes to clinically evaluated compounds. This approach has already shown success in multiple systems. For example, in vivo inhibition of the HSV helicase-primase complex reduces viral replication, while inhibition of the HCV NS3 helicase suppresses replication in cell culture. Advanced computational and fragment-based screens of the SARS-CoV-2 nsp13 helicase have shown specific, high-affinity binding sub-pockets at both the ATP and RNA binding channels [11,71]. The HSV helicase–primase complex provides the most advanced example to date, with optimized inhibitors demonstrating in vivo antiviral efficacy and reduced viral shedding in clinical studies [56].

However, viral helicases as targets also have some challenges. Because there are many mechanistic similarities between viral and human helicases, unselective inhibitors could pose a risk of toxicity. ATP-competitive inhibitors can potentially inhibit other ATP-dependent proteins. Also, helicases are highly dynamic enzymes that undergo large-scale domain rearrangements during ATP binding and hydrolysis. Therefore, choosing an inhibitory state in helicases is more challenging than targeting the relatively inelastic protease active sites. Moreover, helicases are prone to drug resistance. It has been shown that mutations in the Walker loops, motif VI, or nucleic-acid binding clefts can reduce the inhibitory effect of drugs in HCV and coronaviruses [9,32]. These constraints have directed helicase drug discovery toward virus-specific complexes and nonconserved allosteric or conformationally selective inhibitory mechanisms. In general, helicases have both advantages and potentially disadvantages as antiviral drug targets. Their primary roles in viral life cycles and conserved ATP/RNA-binding sites make them a suitable target for inhibition, while their similarities with host cell enzymes can escalate toxicity risks; the dynamic conformation complicates drug design, and resistance mutations can render inhibitors ineffective.

### 5.2. Antiviral Helicase Inhibitors

Several classes of direct-acting inhibitors have shown helicase-inhibitory effects. Guanidine hydrochloride (GuHCl) was an early antiviral compound that blocked picornavirus replication by inhibiting the ATPase activity of 2C [51]. Targeted antiviral methods for coronaviruses emerged; for instance, bananins, which are a group of polyhydroxy compounds derived from adamantane, blocked the ATPase activity of the SARS-CoV helicase and ceased the replication of the virus in cell culture. Natural flavonoids such as scutellarein and myricetin directly deactivate the SARS helicase nsp13 by binding to its nucleotide-binding site and preventing ATP hydrolysis and nucleic acid unwinding [8]. In HCV, several chemical groups, including acridines, quinoline derivatives, and tropolones, were tested against the NS3 helicase. Despite having promising inhibition in vitro at micromolar concentrations, few were developed further because of cytotoxicity or inadequate selectivity [72,73]. These analyses on helicases show that although direct helicase inhibition is feasible, it carries significant challenges.

Viral helicase inhibitors are commonly categorized as ATP-site inhibitors, allosteric conformational traps, complex-specific inhibitors, or repurposed/pleiotropic scaffolds [3,74]. Beyond guanidine hydrochloride, early benzimidazole compounds such as 2-(α-hydroxybenzyl)-benzimidazole (HBB) provided initial evidence that picornavirus 2C is a druggable replication factor, with resistance mapping to single amino-acid substitutions in 2C [75]. Subsequent benzimidazole derivatives, including MRL-1237 and TBZE-029, showed activity against all three poliovirus serotypes, consistent with a shared 2C-dependent mechanism [76,77]. More recent screening and repurposing efforts identified structurally diverse inhibitors, such as dibucaine, zuclopenthixol, pirlindole, hydantoin derivatives, and fluoxetine that suppress replication by engaging conserved or allosteric regions of the 2C ATPase and stabilizing nonproductive conformational states [78]. Overall, these findings show that 2C can be inhibited as an ATP-driven target, even though clear duplex-unwinding activity remains difficult to demonstrate in vitro [50].

In flaviviruses, the NS3 helicase has been directly targeted through inhibition of its ATPase and strand-unwinding activities [63]. Among the compounds with antiviral activity, novobiocin and the more recently described compound 71 (6-bromo-1,2-naphthalenedione) have shown efficacy in ZIKV infection models [79]. Additional direct NS3 helicase inhibitors include benzothiazole-based compounds, such as ML283 analogues, and pyrrolone derivatives, which inhibit ATP hydrolysis and RNA unwinding and reduce DENV and WNV replication in cell-based assays [80]. Earlier chemical scaffolds, including acridine, quinoline, and tropolone derivatives, were also shown to inhibit NS3 helicase activity in vitro but were not broadly advanced further due to cytotoxicity, limited selectivity, or unclear mechanisms of action [72,81]. Repurposed compounds such as suramin have also been reported to inhibit flaviviral NS3 helicase activity in vitro [82].

Unlike most viral helicases, which have only been explored at the preclinical level, the HSV UL5-UL52-UL8 helicase-primase complex has produced inhibitors that are effective in vivo and have been clinically validated [56]. Early high-throughput screens identified T157602, a 2-aminothiazole compound that inhibits both helicase and primase activity [83]. Additional medicinal chemistry work produced thiazolylphenyl-based viral helicase inhibitors such as BILS 179 BS, which showed strong oral efficacy in mouse infection models and remained effective when treatment was started after infection. Related inhibitors, including BILS 22 BS and BILS 45 BS, also remained active against acyclovir-resistant HSV strains [84,85]. This inhibitor series eventually reached the clinic. Pritelivir (BAY 57-1293) showed nanomolar activity against HSV and, in Phase II studies, led to a clear reduction in HSV-2 shedding [86]. Amenamevir (ASP2151), which acts on the same helicase–primase target but has broader activity across alphaherpesviruses, is now approved in Japan for herpes zoster [87]. More recent compounds, such as IM-250 (adibelivir), show higher potency, retain activity against resistant viruses, and display improved penetration into neural tissues, with effects on viral reactivation reported in preclinical models [88]. Overall, these findings make HSV helicase–primase inhibition the most clinically validated example of successful antiviral targeting of helicase function [57].

Direct inhibitors of coronavirus nsp13 include the flavonoids myricetin and scutellarein, which inhibit ATPase activity, and bananin compounds, which act as noncompetitive ATPase inhibitors [89,90]. The triazole compound SSYA10-001 inhibits nsp13 helicase unwinding and reduces replication of SARS-CoV, MERS-CoV, and murine hepatitis virus [91]. High-throughput screening identified FPA-124 as a detergent-insensitive inhibitor that suppresses both NTPase and helicase activities and decreases SARS-CoV-2 replication in infected cells [92]. Suramin and related polysulfonated compounds (NF-023, PPNDS, Evans Blue, Diphenyl Blue) inhibit nsp13 activity but show broad, nonspecific effects [92]. Structure-guided studies identified chromone-4c and related chromone scaffolds that bind near the ATP-binding region and disrupt helicase function [93]. Ranitidine bismuth citrate and other bismuth salts inhibit nsp13 ATPase and helicase activities and reduce viral replication in vivo [94]. Additional inhibitors include diketo acid derivatives, which inhibit nsp13 through ATP-independent mechanisms, and the repurposed compound IOWH-032, which interferes with RNA binding [95]. More recently, direct biochemical screening has identified a broader set of flavonoid-based inhibitors, including quercetin, kaempferol, baicalein, flavanone, flavanone-7-O-glucoside, diosmetin, prunetin, wogonin, dihydromyricetin, and licoflavone C, the latter uniquely inhibiting both ATPase and unwinding activities [96]. Compounds such as posaconazole, grazoprevir, silver sulfadiazine, and plicamycin have been proposed as nsp13 inhibitors in repurposing or virtual screens but remain at a preclinical validation stage [97].

Research is ongoing, for example, the Heli-SMACC database, which is a collection of over 20,000 entries on the interactions between helicases and various biological substances and has enabled scientists to identify potential drug candidates (Table 2) [71]. Several chemical families have been identified through fragment-based screens against SARS-CoV-2 nsp13 and high-throughput approaches that affect the ATPase or unwinding activity of the SARS-CoV-2 nsp13 protein [7]. Potential antivirals bind to allosteric pockets rather than conserved ATP sites, which leads to better selectivity of the compound. The fact that all of these compounds have failed to reach late-stage development shows that in vitro inhibition does not guarantee clinical effectiveness [98,99].

### 5.3. Screening and Lead Optimization Strategies

The discovery of helicase inhibitors has long been hindered by technical limitations. Adapting standard unwinding assays for smaller formats was not easy, which made them unsuitable for large-scale or high-throughput screens [3,100]. Fluorescence-based ATPase assays and FRET (Förster resonance energy transfer)-based strand displacement methods have now overcome these challenges, enabling the real-time and high-throughput analysis of helicase activity [7]. Importantly, these methods make drug discovery more feasible by enabling rapid screening and direct comparison of helicase inhibitors, while also clarifying how individual compounds affect unwinding activity, ATP hydrolysis, and their functional coupling [101]. The latest progress in structural biology has been very helpful as well. For instance, the dynamic ATP-driven conformational cycles and new allosteric binding sites of helicases such as SV40 large T antigen and SARS-CoV-2 nsp13 have been revealed through high-resolution cryo-EM and crystallography methods. By revealing multiple conformational states and binding interfaces of helicases, these structures provide a structural basis for identifying non-ATP-site regions that can be targeted by small-molecule inhibitors [31]. Through computational methods such as Gaussian accelerated molecular dynamics (GaMD), researchers have simulated how helicases undergo conformational changes and can predict stable inhibitor binding modes [32]. These breakthroughs are highly beneficial for rational drug design. Moreover, cheminformatics platforms such as Heli-SMACC now facilitate scaffold mining and cross-family activity prediction, connecting in silico prioritization with in vitro screening. By organizing helicase bioactivity data into a searchable resource, Heli-SMACC enables informed selection of candidate compounds for experimental testing and reduces reliance on blind screening [71]. Altogether, these novel approaches show how the process of drug discovery against helicases has transformed from mostly empirical biochemical testing to a more organized approach based on the drug target’s structure.

### 5.4. Resistance Mechanisms in Viral Helicases

Like other viral enzymes, helicases can develop resistance to drugs. For example, mutations in ATP-binding loops and RNA-binding clefts of the HCV NS3 can minimize the inhibitory effect of helicase-targeting compounds [102]. Coronavirus nsp13 is likely to develop drug-resistant mutations in its motifs Ia, IV, and VI regions under selective pressure [62]. Resistance mechanisms have also been shown to be prevalent in enteroviral helicases. As an example, in poliovirus 2C protein, guanidine-resistant variants show mutations such as N179G and M187L near the Walker B motif, while hydantoin-resistant viruses have F190L and I227V mutations [103]. The dynamic structure of helicases may enable them to tolerate amino acid changes while maintaining function, a feature that provides them with multiple potential escape mechanisms. Approaches to overcome resistance involve both drug design and treatment strategy. Pharmaceutical chemistry efforts are now shifting towards virus-specific allosteric pockets outside the conserved ATP-binding cleft to improve selectivity, although these regions can still accumulate resistance mutations. Therapeutically similar to HIV and HCV treatments, the combination of helicase inhibitors with polymerase or protease drugs decreases the probability of resistance. Viral resistance can be prevented by targeting host factors; however, toxicity concerns outweigh this advantage. Overall, this resistance in viral helicases can be managed through combination therapies or inhibitors with resistance-tolerant binding designs.

## 6. Conclusions

Viral helicases are crucial for genome replication, gene expression, and virion assembly, making them highly promising yet complex targets for antiviral therapeutics. Despite their structural diversity across many helicase superfamilies, shared mechanistic principles, most importantly conserved ATP-dependent translocation modes and functional coupling to viral partner proteins and replication complexes, create several regulatory points that can be pharmacologically targeted. However, such structural conservation may also constrain therapeutic selectivity and facilitate resistance in certain pathological contexts, indicating that conservation alone does not necessarily enhance druggability. Advances in structural biology, computational modeling, and high-throughput screening have expanded opportunities for structure-guided inhibitor discovery, although challenges such as conformational flexibility, similarity to host enzymes, and the rapid emergence of resistance remain substantial obstacles. Overall, a more detailed understanding of viral helicase mechanisms in their native replication contexts will be necessary for guiding next-generation antiviral strategies and for establishing these enzymes as practical therapeutic targets.

## Figures and Tables

**Table 2:** Representative chemical structures of viral helicase inhibitors.

Inhibitor/Class	Structure	Target Helicase (Virus/Host)
Guanidine hydrochloride (GuHCl)	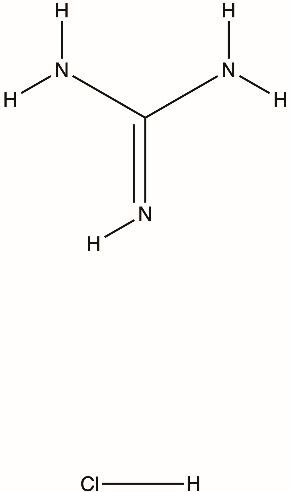	2C helicase, picornaviruses (e.g., poliovirus)
Bananins (adamantane-derived polyhydroxy compounds)	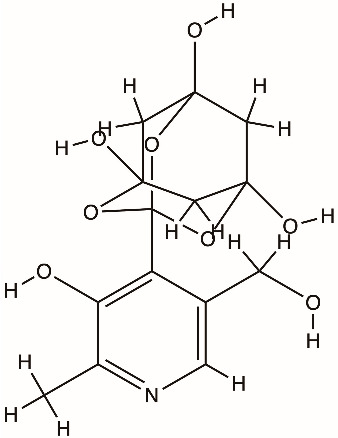	nsp13 helicase, SARS-CoV
Scutellarein (flavonoid)	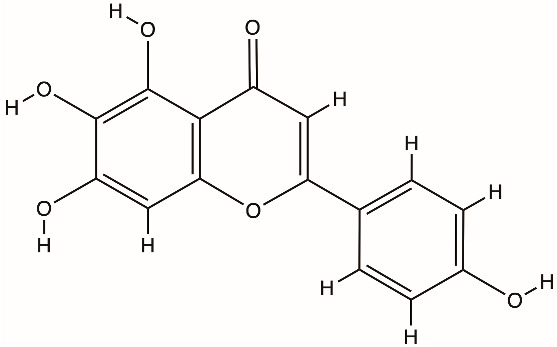	nsp13 helicase, SARS-CoV
Myricetin (flavonoid)	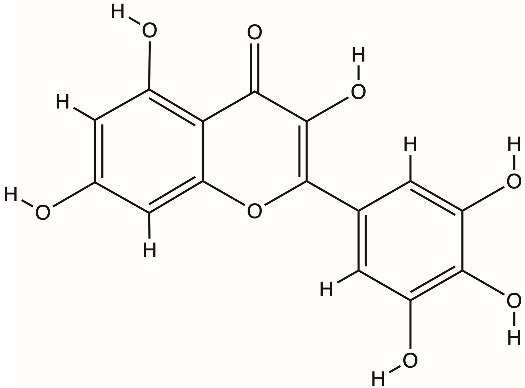	nsp13 helicase, SARS-CoV
Acridines	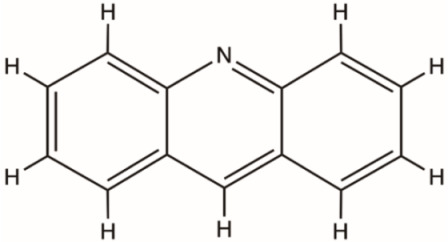	NS3 helicase, HCV
Acridones	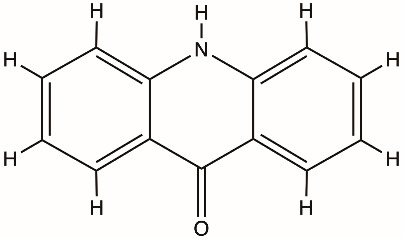	NS3 helicase, HCV
Quinoline derivatives	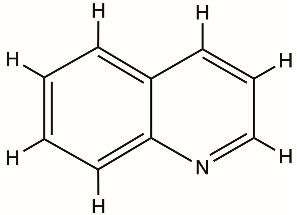	NS3 helicase, HCV
Tropolones	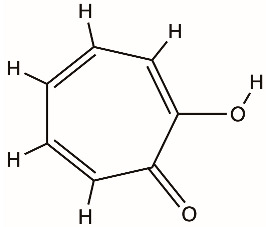	NS3 helicase, HCV

## Data Availability

Data is contained within the article.

## Abbreviations

The following abbreviations are used in this manuscript:

AAA+	ATPases associated with diverse cellular activities
AAV	Adeno-associated virus
Cryo-EM	Cryogenic electron microscopy
DDX	DEAD-box helicase family
dsDNA	Double-stranded DNA
dsRNA	Double-stranded RNA
E1	HPV E1 helicase
GaMD	Gaussian accelerated molecular dynamics
GuHCl	Guanidine hydrochloride
HBV	Hepatitis B virus
HCV	Hepatitis C virus
HIV-1	Human immunodeficiency virus type 1
HSV	Herpes simplex virus
IFN-β	Interferon-beta
NTP	Nucleoside triphosphate
NTPase	Nucleoside triphosphatase
NS3, NS4A, NS4B	Flaviviral nonstructural proteins
nsp13	Coronavirus nonstructural protein 13
PAMP	Pathogen-associated molecular pattern
P-body	Processing body
RTC	Replication–transcription complex
RTPase	RNA 5′-triphosphatase
SF1–SF6	Helicase superfamilies 1–6
ssDNA	Single-stranded DNA
ssRNA	Single-stranded RNA
SV40	Simian virus 40
TRIF	TIR-domain-containing adapter-inducing interferon-β
UTR	Untranslated region
WNV	West Nile virus
ZBD	Zinc-binding domain
